# The autonomic activity of nightmare sufferers during sleep and emotion-evoking picture viewing

**DOI:** 10.1192/j.eurpsy.2022.910

**Published:** 2022-09-01

**Authors:** V. Tomacsek, B. Blaskovich, R. Reichardt, P. Simor

**Affiliations:** 1 Eötvös Loránd University, Institute Of Psychology, Budapest, Hungary; 2 Max Planck Institute of Psychiatry, Department Of Translational Research In Psychiatry, Munich, Germany; 3 Budapest University of Technology and Economics, Department Of Cognitive Science, Budapest, Hungary; 4 Université Libre de Bruxelles, Neuropsychology And Functional Neuroimaging Research Unit At Center For Research In Cognition And Neurosciences And Uni - Ulb Neurosciences Institute, Bruxelles, Belgium

**Keywords:** heart rate variability, emotion regulation, nightmare disorder, parasympathetic regulation

## Abstract

**Introduction:**

In nightmare disorder, dysfunctional emotion regulation goes along with poor subjective sleep quality, which is characterised by pathophysiological features such as abnormal arousal processes and sympathetic influences. Dysfunctional parasympathetic regulation, especially before and during REM phases, is assumed to alter heart rate (HR) and its variability (HRV) of frequent nightmare recallers.

**Objectives:**

We hypothesised that cardiac variability is attenuated in participants experiencing frequent nightmares as opposed to healthy control subjects during less deep sleep stages and an emotion-evoking picture-rating task.

**Methods:**

Based on the second-nights’ polysomnographic recordings of 24 nightmare disordered (NM) and 30 control (CTL) subjects, we examined HRV during pre-REM, REM, post-REM and slow wave sleep periods, separately. Additionally, ECG recordings of wakeful periods such as resting state before sleep onset and an emotional picture-rating task were also analysed.

**Results:**

According to our results, a significant difference was found in the HR of the NM and CTL groups in the nocturnal segments but not during resting wakefulness before sleep onset, suggesting autonomic dysregulation, specifically during sleep in nightmare disorder. However, despite the accelerated HR of NM subjects at night, they did not exhibit lower HRV. Regarding the emotional task, we also found a contrast between the NM and CTL subjects’ HR and HRV, which might indicate altered processes of emotion regulation in nightmare disorder, but the two groups’ subjective picture ratings did not differ.

**Conclusions:**

In summary, our study suggests that there might be some trait-like autonomic changes during sleep, but also state-like autonomic responses to emotion-evoking pictures in nightmare disorder.

**Disclosure:**

No significant relationships.

